# Role of Tight Glycemic Control during Acute Coronary Syndrome on CV Outcome in Type 2 Diabetes

**DOI:** 10.1155/2018/3106056

**Published:** 2018-10-04

**Authors:** Ferdinando Carlo Sasso, Luca Rinaldi, Nadia Lascar, Aldo Marrone, Pia Clara Pafundi, Luigi Elio Adinolfi, Raffaele Marfella

**Affiliations:** ^1^Department of Medical, Surgical, Neurological, Metabolic and Aging Sciences, University of Campania, Naples, Italy; ^2^School of Life and Health Sciences, Aston University, Birmingham, UK

## Abstract

Both incidence and mortality of acute coronary syndrome (ACS) among diabetic patients are much higher than those among nondiabetics. Actually, there are many studies that addressed glycemic control and CV risk, whilst the literature on the role of tight glycemic control during ACS is currently poor. Therefore, in this review, we critically discussed the studies that investigated this specific topic. Hyperglycemia is implicated in vascular damage and cardiac myocyte death through different molecular mechanisms as advanced glycation end products, protein kinase C, polyol pathway flux, and the hexosamine pathway. Moreover, high FFA concentrations may be toxic in acute ischemic myocardium due to several mechanisms, thus leading to endothelial dysfunction. A reduction in free fatty acid plasma levels and an increased availability of glucose can be achieved by using a glucose-insulin-potassium infusion (GIKi) during AMI. The GIKi is associated with an improvement of either long-term prognosis or left ventricular mechanical performance. DIGAMI studies suggested blood glucose level as a significant and independent mortality predictor among diabetic patients with recent ACS, enhancing the important role of glucose control in their management. Several mechanisms supporting the protective role of tight glycemic control during ACS, as well as position statements of Scientific Societies, were highlighted.

## 1. Introduction

Diabetes has become one of the main causes of morbidity and mortality in most countries. It is estimated that 346 million people worldwide have diabetes, and its incidence is arising.

According to the WHO [http://www.who.int/diabetes/en/], diabetes is predicted to become, by 2030, the seventh leading cause of death in the world. Cardiovascular disease represents one of the major complications of diabetes and is responsible for 50% to 80% of early deaths.

A large Danish population-based study conducted on 3.3 million people showed, in diabetic patients requiring glucose-lowering therapy, a cardiovascular risk comparable to nondiabetics who suffered from acute coronary syndrome (ACS), due to which these kinds of patients should receive intensive primary prevention for CVD (antiplatelet therapy, statins, and angiotensin-converting enzyme inhibitor/angiotensin receptor blocker) [1].

The MONICA study also showed a higher incidence of ACS among diabetic patients rather than nondiabetics and that, overall, ACS mortality is four times higher in a male and seven times higher in a female diabetic population [2]. Moreover, a linear positive relationship between admission hyperglycemia and mortality after ACS has been reported. However, in this setting of patients, the optimal management goal of glucose levels still remains uncertain.

The aim of this brief review is to assess the role of a tight glycemic control during ACS in type 2 diabetic subjects, independently from other therapies commonly used for ACS (e.g., statins, antithrombotic therapies, and drug-eluted stents).

## 2. Rationale for the Goal of Glycemic Control in Diabetic Population

Three studies, ADVANCE [3], ACCORD [4], and VADT [5], have reported unremarkable effects of an intensive glucose lowering on cardiovascular events and overall mortality in type 2 diabetes. Indeed, these trials showed that an intensive therapy performed to gain a too low HbA1c target seems to increase the CV risk. Moreover, an intensive antihyperglycemic therapy increased the risk of severe hypoglycemia.

On the other hand, the UKPDS [6] did not demonstrate a significant reduction of macrovascular events during the intensive treatment, whilst showing that the benefits of an intensive strategy to control blood glucose levels appeared 10 years after the end of treatments [7]. It is outstanding that the UKPDS study population was, with respect to ACCORD, ADVANCE, and VADT studies, younger, with less history of CV disease and neuropathy, lower baseline HbA1c, and lower risk of hypoglycemia.

Actually, in a more recent metaregression analysis [8], a higher BMI, duration of diabetes and incidence of severe hypoglycemia revealed to be associated with a greater risk of cardiovascular death in intensive treatment groups. The same meta-analysis showed that an intensified hypoglycemic treatment in type 2 diabetic patients leads to a significant reduction of the incidence of myocardial infarction, whilst not affecting the incidence of stroke and cardiovascular mortality.

All these findings suggest that the HbA1c target should be set based on the phenotype of diabetic patients like a dress. The International Scientific Society Guidelines have accepted this evidence in order to reduce the CV risk among diabetic people [9].

Unfortunately, less evidences are present for what concerns the impact of a tight glycemic control during acute ischemic events on the short- and long-term CV outcome.

Recent RCTs, which have showed the efficacy of some SGLT2-i and GLP-1 RA (empagliflozin, canagliflozin, and liraglutide) to significantly reduce the CV events in diabetics with history of CVD or at very high CV risk, have a great clinical impact. Moreover, empagliflozin and liraglutide reduced the CV mortality among diabetic people in secondary CV prevention [10–12]. Actually, these findings were not applicable on subjects in primary CV prevention and, above all, in patients with ACS. Within the end of 2018, we expect the results of the DECLARE study, whose aim was to assess the CV effect of dapagliflozin on diabetic patients and also on the primary CV prevention (60% of enrolled population) [13].

Actually, non-RCT showed a protective CV effect by the other classes of antihyperglycemic agents.

In fact, RCTs on DPP4i (saxagliptin, alogliptin, and sitagliptin) showed a noninferiority for the primary endpoint of a composite of death from cardiovascular causes, nonfatal myocardial infarction or nonfatal stroke [14–16]. In particular, alogliptin was originally used in diabetic patients with either acute myocardial infarction (AMI) or unstable angina requiring hospitalization within the previous 15 to 90 days [15]. Moreover, saxagliptin showed an increased rate of heart failure hospitalization [14].

Instead, the addition of empagliflozin and of canagliflozin [10, 11] to the standard of care led to a significant reduction in the hospitalization rates for heart failure compared with placebo (35% and 33%, respectively).

Fascinatingly, the PROactive study showed a not statistically significant 10% reduction in the primary composite endpoint (a combination of cardiovascular disease-driven and procedural events in all vascular beds) versus the statistically significant 16% decrease in the main secondary endpoint (all-cause mortality, myocardial infarction, and stroke) observed with pioglitazone in the secondary CV prevention [17]. Recently, the TOSCA.IT study [18], a long-term, pragmatic trial, showed a similar incidence of cardiovascular events with sulfonylureas and pioglitazone as add-on treatments to metformin.

Because of these and many other evidences, we proposed to critically discuss the literature data regardless of the antihyperglycemic agent used, only selecting the few studies which investigated a strict glycemic control during an ACS.

## 3. Diabetic Patients after ACS Are at Very High CV Risk

Diabetic patients experience a higher in-hospital mortality and postinfarction complications than nondiabetic ones, such as heart failure, atrial fibrillation, conduction abnormalities, and angina. The poorer outcome among diabetic patients with AMI does not appear to be explained by a larger infarct size. The delayed improvement of both ventricular performance and metabolic disorders at the noninfarcted area level may be responsible for these adverse outcomes, along with an underlying cardiac dysfunction [19].

Many risk factors are involved in the ACS development and progression among which are metabolic syndrome, insulin resistance, hyperglycemia, and oxidative stress [20]. Anyway, a clear understanding of the pathophysiologic mechanisms underlying the infarcted diabetic heart is still missing.

In diabetic patients, metabolic syndrome is associated with a prothrombotic state, involving endothelial dysfunction, hypercoagulability, and a reduced response to fibrinolysis. These complex mechanisms seem to be related to a decreased functional performance of the ischemic organs and a decreased success of both acute and long-term intervention strategies [21]. Among the metabolic risk factors, atherogenic dyslipidemia, associated with an increased number of small dense low-density lipoproteins (LDL), appears to play a predictive role either in the development of cardiovascular events or in the progression of coronary artery disease (CAD) in diabetic patients [22].

## 4. Role of Insulin Resistance in ACS

Significant evidence supports the theory of a strict relationship between insulin resistance and cardiovascular disease [23]. The insulin effects on inflammatory response, vascular tone, and angiogenesis are attributable to an increased synthesis of nitric oxide and are deeply reduced in the insulin-resistant states. Insulin infusion, with algorithms aiming to provide an optimal blood glucose control, improves the clinical outcomes of patients with severe acute illness and ACS [24]. Insulin resistance causes a progressive endothelial dysfunction and modifications of glucose and lipid metabolism, establishing a continuous negative feedback cycle and eventually leading to an acute vascular damage [23]. Actually, both insulin resistance and hyperglycemia seem to play important roles in the pathogenesis of ACS.

## 5. Effects of Hyperglycemia during ACS

Several studies identified hyperglycemia as an independent risk factor for diabetic cardiomyopathy, through cardiac cell apoptosis [25]. Apoptotic myocyte loss could represent an important mechanism leading to a poor prognosis after AMI in diabetic patients as it contributes to a progressive cardiac remodeling, through left ventricular enlargement and interstitial fibrosis, resulting in an increased synthesis of type III collagen by cardiac fibroblasts [26].

Abnormal glucose tolerance is almost twice among patients with an ACS, as in population-based controls [27]. Hyperglycemia acts as a multiplier of cardiovascular risk and is implicated in vascular damage and cardiac myocyte death through different molecular mechanisms: advanced glycation end products (AGE), protein kinase C (PKC), polyol pathway flux, and the hexosamine pathway. All of these reflect a single hyperglycemia-induced process of overproduction of superoxide by the mitochondrial electron transport chain [20].

More fascinatingly, in a high-risk intensive cardiac care unit general population (only 17% had known diabetes), both hyperglycemia at admission (glucose ≥ 9 mmol/L) and sustained hyperglycemia during hospitalization (average glucose levels ≥ 8 mmol/L) were independent predictors of all-cause mortality [28]. Similar relationships between admission glucose levels and hospital mortality were also reported in other studies [29].

ACS results in many systemic metabolic changes, particularly evident in diabetic patients with an already reduced capability of insulin secretion and use of glucose for the production of energy. Clinical and experimental evidences suggest that the sympathoadrenal activation contributes to mortality in patients with ischemic heart disease and the magnitude of the adrenocortical response is governed by the amount of myocardial necrosis [30]. An excessive catecholamine activity, through a glycogenolytic effect, contributes to a rise in blood glucose levels. In addition, adrenaline is a powerful suppressor of the normal insulin response to a glucose load.

The main result of these hormonal pathways is an increased turnover of FFAs. In well-oxygenated hearts, FFAs have been identified as the preferred substrate by both *in vivo* and *in vitro* studies, accounting for 35% to 75% of oxygen consumption. In hypoxic hearts, FFA oxidation is suppressed and glycolysis stimulated, leading to an increase of triglyceride levels. Experimental and clinical observations suggest that increased circulating concentrations of FFAs may be associated with an adverse outcome of ACS [31], by means of several mechanisms such as direct toxicity, increased oxygen demand, and direct inhibition of glucose oxidation. These metabolic changes may play a role in the development of arrhythmias and disorders of conduction. This relationship could be explained by two mechanisms: stimulation of the hypoxic myocardium by increased circulating catecholamine and increased myocardial oxygen requirement resulting from the utilization of FFAs as an energy substrate [32].

Recently, several mechanisms showed how high FFA concentrations may be toxic in acute ischemic myocardium, such as mitochondrial uncoupling, activation of lipids in the mitochondria, inhibition of *β*-oxidation, inhibition of the Na^+^-K^+^-ATPase pump leading to high intracellular sodium and calcium, or GLU-4 reduction causing reduced insulin-stimulated glucose transport [32]. Thus, monitoring and reducing concentrations of FFAs during and after an ACS represent a priority [33].

Recently, it was confirmed that the FFA level might be a predictor of the severity of myocardial ischemia during the subacute onset of ACS attack and was observed that the FFA levels increased with the severity of necrosis and ischemia, such as cTnT [31]. In the same paper, an association between WBC counts, hs-CRP, and FFA levels was observed in ACS, suggesting a possible mechanism relating FFAs together with inflammatory factors affecting the progress of ischemia. Moreover, elevated circulating FFA levels led to endothelial dysfunction *in vivo* through the activation of PKC-mediated inflammatory pathways and an excessive generation of oxidants [34, 35], which would partially explain a proarrhythmogenic activity of FFAs.

## 6. Are There Clinical Evidences for Tight Glycemic Control during ACS?

A reduction in free fatty acid plasma levels and an increased availability of glucose can be achieved by using a glucose-insulin-potassium infusion (GIKi) during AMI. The GIKi is associated with an improvement of either long-term prognosis or left ventricular mechanical performance [36]. Moreover, early after AMI, high-dose GIK infusion improves the cardiac function, as confirmed by hemodynamic measurements. In fact, high-dose GIK can decrease the cardiomyocyte apoptosis in AMI patients with reperfusion therapy [37]. Moreover, high-dose GIK could improve cardiac remodeling in AMI patients receiving primary PCI by lowering vascular resistance [38].

As a confirmation of this hypothesis, the DIGAMI study (Diabetes Mellitus, Insulin Glucose Infusion in Acute Myocardial Infarction) showed that GIKi administration in the early 24 hours after acute myocardial infarction (AMI), followed by a multidose subcutaneous insulin regimen, facilitates a persistent improvement of glucose control and reduces the long-term mortality in diabetic patients. In particular, the relative mortality reduction reduced by 29% after 1 year. Interestingly, this particularly manifested in patients with a low cardiovascular risk profile and no previous insulin treatment [39]. Actually, the DIGAMI study has brought too many criticisms, such as the uncertainty whether the GIK infusion during AMI or the followed long-term insulin treatment caused the favorable long-term outcome, the small simple size, large confident intervals and the potential bias resulted with only the 50% of all eligible patients being randomized.

The DIGAMI 2 trial was planned and conducted to further investigate the possible effects on mortality and morbidity of an insulin-based management on diabetic patients with AMI. In this trial, three treatment strategies were compared: acute insulin-glucose infusion followed by insulin-based long-term glucose control; insulin-glucose infusion followed by standard glucose control and routine metabolic management according to local practice. The study did not confirm the usefulness on the overall survival rate due to an early and long-term insulin treatment in type 2 diabetic patients following AMI. In fact, neither an acutely introduced long-term insulin treatment did not improve survival in type 2 diabetic patients following myocardial infarction when compared with a conventional management at similar levels of glucose control nor an insulin-based treatment lowers the number of nonfatal myocardial reinfarctions and strokes [40]. In particular, DIGAMI 2 did not show any mortality benefit in a maximum follow-up time of up to 3 years. However, these results suggested blood glucose levels as a significant and independent mortality predictor among diabetic patients, enhancing the important role of glucose control in their management.

Moreover, a post hoc analysis of DIGAMI 2 [41], adjusting for confounders such as glycemic control, did not show any significant difference in mortality among sulphonylureas, metformin, and insulin. However, the risk of nonfatal myocardial infarction and stroke was significantly increased by insulin treatment, whilst metformin was protective.

The most reasonable reason for the difference between DIGAMI 1 and 2 findings is that in DIGAMI 2, changes in glucose concentrations between control and insulin treatment groups were nonsignificant, despite the intent to obtain target-driven, strict glycemic control in patients assigned to the insulin-based groups in these trials. Moreover, HbA1c at admission was substantially higher in DIGAMI 1 than in DIGAMI 2 (HbA1c 8.2% vs 7.2%). Interestingly, findings from the recent 20-year follow-up of the DIGAMI 1 cohort supported that insulin-based intensified glycemic control after acute myocardial infarction increased survival, with a lasting effect of at least 8 years [42]. In particular, contrarily to the favorable effects observed in patients with no previous insulin use and at a low cardiovascular risk, in whom longevity was prolonged most, from 6.9 years to 9.4 years, intensified insulin-based glycemic control did not affect the outcome in patients at high risk and no previous insulin treatment. These findings seem to support the conclusions from the ACCORD and ADVANCE trials, which demonstrated that a tight glycemic control in patients with long-standing diabetes and advanced cardiovascular disease does not improve mortality.

## 7. Mechanisms Supporting the Protective Role of Tight Glycemic Control during ACS

Accumulating evidence supports the hypothesis that the heart has a pool of cardiac stem-progenitor cells (CSCs), which can differentiate into cardiomyocytes and acutely populate the damaged regions of ischemic myocardium, regenerating coronary vessels [43]. In particular, Anversa and coworkers proposed a classification of cardiac immature cells into 4 classes: cardiac stem cells (CSCs), progenitors (CPCs), precursors (MPCs), and amplifying cells. These cell types may be considered as subsequent steps in the progressive evolution from a more primitive to a more differentiated phenotype [44].

There is evidence that diabetes plays an important role in the dramatic loss of MPC function in animal models. The high levels of oxygen reactive species, produced by hyperglycemia during AMI, result in the inhibition of both cell replication and differentiation, thus favoring the development of a cardiac myopathy characterized by a decrease in muscle mass and impaired ventricular function [45]. Both MPC number and myocyte proliferation significantly increase when a tight glycemic control is achieved in the early stage of AMI. A tight glycemic control during an acute ischemic damage is associated with an increased regenerative potential of the myocardium [31]. Glucose control may have more important results than insulin treatment in the improvement of the cardiac outcome among diabetic patients.

In 2011, Samaropoulos and coworkers demonstrated how an intensive glycemic control in middle- to old-aged type 2 diabetic patients, who already had or are at risk for cardiovascular disease, was associated with a reduction in high-sensitivity C-reactive protein (hs-CRP) [46].

This finding suggests that an increased inflammatory immune process seems to be most likely a mechanism linking acute hyperglycemias to poor cardiac outcomes in AMI patients [47]. Inflammatory response and cytokine elaboration are particularly active after AMI and contribute to cardiac remodeling, through progressive myocyte apoptosis, hypertrophy, and defects in contractility [48].

Recently, Tatsch et al. showed an association between a poor control of type 2 diabetes and increased levels of oxidative, inflammatory, and endothelial biomarkers, resulting in DNA damage [49].

High glucose levels have been reported to enhance inducible nitric oxide synthase (iNOS) expression, leading to the production of high levels of nitric oxide (NO) [50]. Moreover, iNOS is expressed in the myocardium after MI. Although NO may have beneficial effects on the inflammatory response and the vascular resistance, increased NO levels contribute to the production of peroxynitrite, hence producing a myocardial damage and a higher mortality after AMI [51].

In addition to hyperglycemia, oxidative stress may be induced by soluble advanced glycation end products (AGE). Among AGE precursors, methylglyoxal (MG) is considered as one of the key intermediates linking hyperglycemia and intensive lipolysis, two dominant metabolic changes in diabetes [52]. Oxidized low-density lipoprotein (oxLDL) in diabetic patients enhances monocyte chemoattractant protein-1 (MCP1) gene expression in endothelial cells, increasing the atherogenic process and promoting endothelial dysfunction [53].

Moreover, deleterious vascular effects of endothelial dysfunction are associated with smooth muscle cell proliferation after vascular injury, including injury from catheter-based interventions. In fact, diabetic patients have a greater incidence of restenosis after percutaneous coronary intervention (PCI), related to an exaggerated tissue proliferation in lesions treated either with or without stents. The restenosis process begins very early, between 1 and 3 months after coronary angioplasty [54]. Timmer and colleagues examined the effects of a periprocedural tight glycemic control during PCI on the restenosis rate in hyperglycemic patients with ST segment elevation myocardial infarction (STEMI), showing that both elevated glucose admission and HbA1c levels are associated with adverse outcomes [55].

## 8. Evidences for Tight Glycemic Control in Surgical and Critically Ill Patients

Several studies, though not conducted in patients with acute coronary syndrome, partially confirmed this relationship between CV outcome and glycemic *milieu*. A tight glycemic control during the perisurgical period seems to decrease the inflammatory immune reaction, as well as both nitrotyrosine levels and MCP1, hence driving to a better prognosis [56]. In reality, insulin resistance during surgery, rather than the presence of diabetes mellitus, is associated with an increased risk of major complications.

It is well known that major surgical tissue trauma leads to alterations in glucose metabolism, resulting in hyperglycemia and insulin resistance. This could be explained by specific neuroendocrine changes, such as increased circulating concentrations of cortisol, glucagon, and catecholamine. The extent of insulin resistance during surgery depends on the intensity of trauma, suggesting insulin resistance as a marker of surgical stress, with potential relevance for the clinical outcome [57]. The number of patients who suffered a major complication and an increased rate of superficial wound infections significantly increased in diabetics with poor preoperative glycemic control when compared with nondiabetics.

Van den Berghe and colleagues obtained similar results studying patients treated in an intensive care unit (ICU) [58]. They asserted that an intensive insulin therapy during intensive care prevents morbidity, though not significantly reducing mortality. More recently, Brunkhorst et al. [59] confirmed that there are no significant differences in the death rate for the insulin intensive-treated group and showed that the use of intensive therapy in critically ill patients determines an increased risk for serious adverse events related to hypoglycemia.

Finally, the NICE-SUGAR study, a large, international, randomized trial [60], suggested that a goal of normoglycemia for glucose control does not necessarily benefit critically ill patients, rather being harmful, due to a major incidence of deaths from cardiovascular causes in the intensive control group compared to that resulted in the conventional control group. Moreover, the former group shows a lower median survival time than the latter. In particular, it was observed that an intensive glucose control increased mortality among adults in ICU: a blood glucose target of 180 mg/dL or less resulted in lower mortality than a target of 81 to 108 mg/dL.

## 9. Position Statements of Scientific Societies

All these studies confirm that hyperglycemia is common during ACS and is associated with increased mortality rates. Anyway, it remains still unclear, as stated by the American Heart Association, whether hyperglycemia is either a marker or a mediator of higher mortality and whether hyperglycemia treatment improves outcomes [61].

Correctly, Scientific Societies, underlying the absence of robust data for an optimal glucose management (e.g., treatment thresholds and glucose targets) in STEMI patients, suggested a close, though not too strict glucose control as that of the best approach.

The last ESC task force on diabetes and CV diseases developed in collaboration with EASD suggested, according to DIGAMI 1, that DM and AMI would benefit from glycemic control, in the case of a significant hyperglycemia (higher than 10 mmol/L or 180 mg/dL), with the target adapted to possible comorbidities as a class 2a recommendation. In particular, an approximation towards normoglycemia with less stringent targets, in those with severe comorbidities, would represent a reasonable goal, though exact targets have still to be defined. Moreover, the two Scientific Societies stated insulin infusion as the most efficient way to rapidly achieve glucose control [62].

More recently, the 2017 ESC Guidelines for the management of acute myocardial infarction in patients presenting with ST segment elevation [63] stated, as class 2a recommendation, that glucose-lowering therapy should be considered in ACS patients with glucose levels > 10 mmol/L (>180 mg/dL), whilst episodes of hypoglycemia (defined as glucose levels ≤ 3.9 mmol/L or ≤70 mg/dL) should be avoided.

Finally, the 2018 ADA guidelines [64] recommended, in hospitalized patients, a glucose target range between 140 mg/dL and 180 mg/dL (7.8–10.0 mmol/L) for the majority of critically ill patients (level A of evidence). Clinical judgment, combined with the ongoing assessment of the patient's clinical status, should be incorporated into the day-to-day decisions regarding insulin doses. Remarkably, the treatment regimen should be reviewed and changed as necessary to prevent further hypoglycemia when a blood glucose value is ≤70 mg/dL (3.9 mmol/L) (level C of evidence).

Notably, whilst all these guidelines do not seem mandatory with regard to a strict hyperglycemic cutoff, instead, they agree to absolutely avoid hypoglycemia. Therefore, according to the recent joint position statement of the American Diabetes Association and the European Association for the Study of Diabetes [65], the hypoglycemia alert value in hospitalized patients has to be defined as blood glucose ≤ 70 mg/dL (3.9 mmol/L) and clinically significant hypoglycemia as glucose values < 54 mg/dL (3.0 mmol/L), to be reported in clinical trials. This statement thus represents a good threshold for future studies.

## 10. Conclusions

In conclusion, optimal glucose target levels and treatment regimens in this setting of patients are still under debate. Future studies on intensive glucose control must be developed to reduce both glucose variability and risk of hypoglycemia, thus achieving an optimal blood glucose concentration in critically ill patients, particularly in ACS.

## References

[1] Schramm T. K., Gislason G. H., Køber L. (2008). Diabetes patients requiring glucose-lowering therapy and nondiabetics with a prior myocardial infarction carry the same cardiovascular risk: a population study of 3.3 million people. *Circulation*.

[2] Lundberg V., Stegmayr B., Asplund K., Eliasson M., Huhtasaari F. (1997). Diabetes as a risk factor for myocardial infarction: population and gender perspectives. *Journal of Internal Medicine*.

[3] The Advance Collaborative Group (2008). Intensive blood glucose control and vascular outcomes in patients with type 2 diabetes. *NEJM*.

[4] The Action to Control Cardiovascular Risk in Diabetes Study Group (2008). Effects of intensive glucose lowering in type 2 diabetes. *NEJM*.

[5] Duckworth W., Abraira C., Moritz T. (2009). Glucose control and vascular complications in veterans with type 2 diabetes. *NEJM*.

[6] UK Prospective Diabetes Study Group (1998). Intensive blood-glucose control with sulphonylureas or insulin compared with conventional treatment and risk of complications in patients with type 2 diabetes (UKPDS 33). *The Lancet*.

[7] Holman R. R., Paul S. K., Bethel M. A., Matthews D. R., Neil H. A. W. (2008). 10-year follow-up of intensive glucose control in type 2 diabetes. *NEJM*.

[8] Mannucci E., Monami M., Lamanna C., Gori F., Marchionni N. (2009). Prevention of cardiovascular disease through glycemic control in type 2 diabetes a meta-analysis of randomized clinical trials. *Nutrition, Metabolism and Cardiovascular Diseases*.

[9] American Diabetes Association (2018). Glycemic targets: standards of medical care in diabetes—2018. *Diabetes Care*.

[10] Zinman B., Wanner C., Lachin J. M. (2015). Empagliflozin, cardiovascular outcomes, and mortality in type 2 diabetes. *The New England Journal of Medicine*.

[11] Neal B., Perkovic V., Mahaffey K. W. (2017). Canagliflozin and cardiovascular and renal events in type 2 diabetes. *The New England Journal of Medicine*.

[12] Marso S. P., Daniels G. H., Brown-Frandsen K. (2016). Liraglutide and cardiovascular outcomes in type 2 diabetes. *The New England Journal of Medicine*.

[13] Wiviott S. D., Raz I., Bonaca M. P. (2018). The design and rationale for the dapagliflozin effect on cardiovascular events (DECLARE)-TIMI 58 trial. *American Heart Journal*.

[14] Scirica B. M., Bhatt D. L., Braunwald E. (2013). Saxagliptin and cardiovascular outcomes in patients with type 2 diabetes mellitus. *The New England Journal of Medicine*.

[15] White W. B., Cannon C. P., Heller S. R. (2013). Alogliptin after acute coronary syndrome in patients with type 2 diabetes. *The New England Journal of Medicine*.

[16] Green J. B., Bethel M. A., Armstrong P. W. (2015). Effect of sitagliptin on cardiovascular outcomes in type 2 diabetes. *The New England Journal of Medicine*.

[17] Dormandy J. A., Charbonnel B., Eckland D. J. (2005). Secondary prevention of macrovascular events in patients with type 2 diabetes in the PROactive study (PROspective pioglitAzone Clinical Trial In macroVascular Events): a randomised controlled trial. *Lancet*.

[18] Vaccaro O., Masulli M., Nicolucci A. (2017). Effects on the incidence of cardiovascular events of the addition of pioglitazone versus sulfonylureas in patients with type 2 diabetes inadequately controlled with metformin (TOSCA.IT): a randomised, multicentre trial. *The Lancet Diabetes and Endocrinology*.

[19] Alegria J. R., Miller T. D., Gibbons R. J., Yi Q. L., Yusuf S., Collaborative Organization of RheothRx Evaluation (CORE) Trial Investigators (2007). Infarct size, ejection fraction, and mortality in diabetic patients with acute myocardial infarction treated with thrombolytic therapy. *American Heart Journal*.

[20] Rahman S., Rahman T., Ismail A. A.-S., Rashid A. R. A. (2007). Diabetes-associated macrovasculopathy: pathophysiology and pathogenesis. *Diabetes, Obesity & Metabolism*.

[21] Durina J., Remkova A. (2007). Prothrombotic state in metabolic syndrome. *Bratislavské Lekárske Listy*.

[22] Rizzo M., Berneis K. (2007). Small dense low-density-lipoproteins and the metabolic syndrome. *Diabetes/Metabolism Research and Reviews*.

[23] Cersosimo E., Defronzo R. A. (2006). Insulin resistance and endothelial dysfunction: the road map to cardiovascular diseases. *Diabetes/Metabolism Research and Reviews*.

[24] Anfossi G., Russo I., Doronzo G., Trovati M. (2007). Relevance of the vascular effects of insulin in the rationale of its therapeutical use. *Cardiovascular & Hematological Disorders Drug Targets*.

[25] Cai L., Li W., Wang G., Guo L., Jiang Y., Kang Y. J. (2002). Hyperglycemia-induced apoptosis in mouse myocardium: mitochondrial cytochrome C-mediated caspase-3 activation pathway. *Diabetes*.

[26] Bäcklund T., Palojoki E., Saraste A. (2004). Sustained cardiomyocyte apoptosis and left ventricular remodelling after myocardial infarction in experimental diabetes. *Diabetologia*.

[27] Bartnik M., Norhammar A., Ryden L. (2007). Hyperglycaemia and cardiovascular disease. *Journal of Internal Medicine*.

[28] Lipton J. A., Barendse R. J., Van Domburg R. T. (2013). Hyperglycemia at admission and during hospital stay are independent risk factors for mortality in high risk cardiac patients admitted to an intensive cardiac care unit. *European Heart Journal Acute Cardiovascular Care*.

[29] Kosiborod M., Rathore S. S., Inzucchi S. E. (2005). Admission glucose and mortality in elderly patients hospitalized with acute myocardial infarction: implications for patients with and without recognized diabetes. *Circulation*.

[30] Zhu D., Xie H., Wang X., Liang Y., Yu H., Gao W. (2015). Correlation of plasma catestatin level and the prognosis of patients with acute myocardial infarction. *PLoS One*.

[31] Ma P., Han L., Lv Z. (2016). In-hospital free fatty acids levels predict the severity of myocardial ischemia of acute coronary syndrome. *BMC Cardiovascular Disorders*.

[32] Pilz S., März W. (2008). Free fatty acids as a cardiovascular risk factor. *Clinical Chemistry and Laboratory Medicine*.

[33] Oliver M. F. (2010). Control of free fatty acids during acute myocardial ischaemia. *Heart*.

[34] Li H., Li H., Bao Y., Zhang X., Yu Y. (2011). Free fatty acids induce endothelial dysfunction and activate protein kinase C and nuclear factor-*κ*B pathway in rat aorta. *International Journal of Cardiology*.

[35] Tang Y., Li G. (2011). Chronic exposure to high fatty acids impedes receptor agonist-induced nitric oxide production and increments of cytosolic Ca^2+^ levels in endothelial cells. *Journal of Molecular Endocrinology*.

[36] Zhao Y. T., Weng C. L., Chen M. L. (2010). Comparison of glucose-insulin-potassium and insulin-glucose as adjunctive therapy in acute myocardial infarction: a contemporary meta-analysis of randomised controlled trials. *Heart*.

[37] Zhang L., Zhang L., Li Y. H. (2005). High-dose glucose-insulin-potassium treatment reduces myocardial apoptosis in patients with acute myocardial infarction. *European Journal of Clinical Investigation*.

[38] Li Y., Zhang L., Zhang L. (2010). High-dose glucose-insulin-potassium has hemodynamic benefits and can improve cardiac remodeling in acute myocardial infarction treated with primary percutaneous coronary intervention: from a randomized controlled study. *Journal of Cardiovascular Disease Research*.

[39] Malmberg K., Rydén L., Efendic S. (1995). Randomized trial of insulin-glucose infusion followed by subcutaneous insulin treatment in diabetic patients with acute myocardial infarction (DIGAMI study): effects on mortality at 1 year. *Journal of the American College of Cardiology*.

[40] Malmberg K., Rydén L., Wedel H. (2005). Intense metabolic control by means of insulin in patients with diabetes mellitus and acute myocardial infarction (DIGAMI 2): effects on mortality and morbidity. *European Heart Journal*.

[41] Mellbin L. G., Malmberg K., Norhammar A., Wedel H., Rydén L., DIGAMI 2 Investigators (2008). The impact of glucose lowering treatment on long-term prognosis in patients with type 2 diabetes and myocardial infarction: a report from the DIGAMI 2 trial. *European Heart Journal*.

[42] Ritsinger V., Malmberg K., Mårtensson A., Rydén L., Wedel H., Norhammar A. (2014). Intensified insulin-based glycaemic control after myocardial infarction: mortality during 20 year follow-up of the randomised diabetes mellitus insulin glucose infusion in acute myocardial infarction (DIGAMI 1) trial. *The Lancet Diabetes and Endocrinology*.

[43] Beltrami A. P., Barlucchi L., Torella D. (2003). Adult cardiac stem cells are multipotent and support myocardial regeneration. *Cell*.

[44] Rota M., LeCapitaine N., Hosoda T. (2006). Diabetes promotes cardiac stem cell aging and heart failure, which are prevented by deletion of the p66^shc^ gene. *Circulation Research*.

[45] Marfella R., Sasso F. C., Cacciapuoti F. (2012). Tight glycemic control may increase regenerative potential of myocardium during acute infarction. *The Journal of Clinical Endocrinology & Metabolism*.

[46] Samaropoulos X. F., Light L., Ambrosius W. T., Marcovina S. M., Probstfield J., Jr D. C. G. (2012). The effect of intensive risk factor management in type 2 diabetes on inflammatory biomarkers. *Diabetes Research and Clinical Practice*.

[47] Marfella R., Siniscalchi M., Esposito K. (2003). Effects of stress hyperglycemia on acute myocardial infarction: role of inflammatory immune process in functional cardiac outcome. *Diabetes Care*.

[48] Nian M., Lee P., Khaper N., Liu P. (2004). Inflammatory cytokines and postmyocardial infarction remodeling. *Circulation Research*.

[49] Tatsch E., Bochi G. V., Piva S. J. (2012). Association between DNA strand breakage and oxidative, inflammatory and endothelial biomarkers in type 2 diabetes. *Mutation Research/Fundamental and Molecular Mechanisms of Mutagenesis*.

[50] Marfella R., Di Filippo C., Esposito K. (2004). Absence of inducible nitric oxide synthase reduces myocardial damage during ischemia reperfusion in streptozotocin-induced hyperglycemic mice. *Diabetes*.

[51] Feng Q., Lu X., Jones D. L., Shen J., Arnold J. M. O. (2001). Increased inducible nitric oxide synthase expression contributes to myocardial dysfunction and higher mortality after myocardial infarction in mice. *Circulation*.

[52] Turk Z., Cavlović-Naglić M., Turk N. (2011). Relationship of methylglyoxal-adduct biogenesis to LDL and triglyceride levels in diabetics. *Life Sciences*.

[53] Panee J. (2012). Monocyte chemoattractant protein 1 (MCP-1) in obesity and diabetes. *Cytokine*.

[54] Armstrong E. J., Rutledge J. C., Rogers J. H. (2013). Coronary artery revascularization in patients with diabetes mellitus. *Circulation*.

[55] Timmer J. R., Hoekstra M., Nijsten M. W. N. (2011). Prognostic value of admission glycosylated hemoglobin and glucose in nondiabetic patients with ST-segment-elevation myocardial infarction treated with percutaneous coronary intervention. *Circulation*.

[56] Cooper M. E., El-Osta A. (2010). Epigenetics: mechanisms and implications for diabetic complications. *Circulation Research*.

[57] Ljungqvist O., Nygren J., Soop M., Thorell A. (2005). Metabolic perioperative management: novel concepts. *Current Opinion in Critical Care*.

[58] van den Berghe G., Wilmer A., Hermans G. (2006). Intensive insulin therapy in the medical ICU. *The New England Journal of Medicine*.

[59] Brunkhorst F. M., Engel C., Bloos F. (2008). Intensive insulin therapy and pentastarch resuscitation in severe sepsis. *The New England Journal of Medicine*.

[60] Finfer S., Chittock D. R., Su S. Y. (2009). Intensive versus conventional glucose control in critically ill patients. *The New England Journal of Medicine*.

[61] Deedwania P., Kosiborod M., Barrett E. (2008). Hyperglycemia and acute coronary syndrome: a scientific statement from the American Heart Association Diabetes Committee of the Council on nutrition, physical activity, and metabolism. *Circulation*.

[62] Authors/Task Force Members, Rydén L., Grant P. J. (2013). ESC Guidelines on diabetes, pre-diabetes, and cardiovascular diseases developed in collaboration with the EASD. *European Heart Journal*.

[63] Ibanez B., James S., Agewall S. (2018). 2017 ESC Guidelines for the management of acute myocardial infarction in patients presenting with ST-segment elevation: the task force for the management of acute myocardial infarction in patients presenting with ST-segment elevation of the European Society of Cardiology. *European Heart Journal*.

[64] ADA–American Diabetes Association (2018). 2018 Standards of diabetes care. *Diabetes Care*.

[65] International Hypoglycemia Study Group (2017). Glucose concentrations of less than 3.0 mmol/L (54 mg/dL) should be reported in clinical trials: a joint position statement of the American Diabetes Association and the European Association for the Study of Diabetes. *Diabetes Care*.

